# Contribution of Nanotechnologies to Vaccine Development and Drug Delivery against Respiratory Viruses

**DOI:** 10.1155/2021/6741290

**Published:** 2021-10-27

**Authors:** Mahdi Ftouh, Nesrine Kalboussi, Nabil Abid, Souad Sfar, Nathalie Mignet, Badr Bahloul

**Affiliations:** ^1^Drug Development Laboratory LR12ES09, Faculty of Pharmacy, University of Monastir, Tunisia; ^2^Sahloul University Hospital, Pharmacy Department, Sousse, Tunisia; ^3^Department of Biotechnology, High Institute of Biotechnology of Sidi Thabet, University of Manouba, BP-66, 2020 Ariana, Tunis, Tunisia; ^4^Laboratory of Transmissible Diseases and Biological Active Substances LR99ES27, Faculty of Pharmacy, University of Monastir, Rue Ibn Sina, 5000 Monastir, Tunisia; ^5^University of Paris, INSERM, CNRS, UTCBS, Faculté de Pharmacie, 4 avenue de l'Observatoire, 75006 Paris, France

## Abstract

According to the Center for Disease Control and Prevention (CDC), the coronavirus disease 2019, a respiratory viral illness linked to significant morbidity, mortality, production loss, and severe economic depression, was the third-largest cause of death in 2020. Respiratory viruses such as influenza, respiratory syncytial virus, SARS-CoV-2, and adenovirus, are among the most common causes of respiratory illness in humans, spreading as pandemics or epidemics throughout all continents. Nanotechnologies are particles in the nanometer range made from various compositions. They can be lipid-based, polymer-based, protein-based, or inorganic in nature, but they are all bioinspired and virus-like. In this review, we aimed to present a short review of the different nanoparticles currently studied, in particular those which led to publications in the field of respiratory viruses. We evaluated those which could be beneficial for respiratory disease-based viruses; those which already have contributed, such as lipid nanoparticles in the context of COVID-19; and those which will contribute in the future either as vaccines or antiviral drug delivery systems. We present a short assessment based on a critical selection of evidence indicating nanotechnology's promise in the prevention and treatment of respiratory infections.

## 1. Introduction

Several viruses were identified in the 20th and 21st centuries. We limit the number of viruses in this review to those linked to major pandemics caused by respiratory viruses (Rvs) that marked these two centuries. In chronological order, five pandemics were caused by influenza viruses: Spanish influenza (1918-1920), Asian flu (1957), Hong Kong flu (1968), Russian flu (H1N1, 1977), and avian influenza H5N1 (1997, 2003 and 2018) [1]. Additionally, two pandemics were caused by two coronaviruses (CoVs): (i) the Severe Acute Respiratory Syndrome CoV (SARS-CoV) originally sprang out around South China in the fall of 2002. After that, it spread to 29 nations or regions [2–4]; (ii) SARS-CoV-2, genetically linked to that of 2002 and animal CoVs, has been detected since December 2019 [5]. Besides the high mortality rate, all of these viruses share several common features; they are RNA viruses and exhibit a remarkable rate of recombination and/or reassortment, with less extent for CoVs, which hampers the development of effective antivirals and specific vaccines. The high mutation rate and recombination/reassortment of several RNA viruses advocate the continued emergence of novel viruses that pose a continued threat to global health and economic systems. Yet, it results in uncontrolled pandemics, such as SARS-CoV-2. Therapeutic antiviral medicines can be utilized as a treatment during the early stages of a disease epidemic when conventional vaccinations are unavailable. Antiviral medicines, on the other hand, should not be cytotoxic and must overcome many obstacles to be effective at a specific location. According to its mechanism of action, its target might be extracellular to block virus/cell receptor interaction or intracellular to hamper the virus replication steps [6]. Additionally, antiviral drugs need to escape from the immune system components and avoid their degradation. Therefore, nanodelivery vehicles could enhance the activity of antiviral drugs and their pharmacokinetic profile while reducing their systemic toxicity. Furthermore, nanotechnology is considered helpful not only to improve the antiviral molecule delivery but also to supply the viral component(s) to the immune system before infection, enhancing the immune response to respond once the infection occurs.

In this review, we will look at how nanotechnology might help in the treatment of respiratory viral infections. We begin with a background in virology, elucidating general characteristics of viruses, emphasizing common features of a specific group of viruses inducing respiratory diseases. The use of nanotechnology to combat viral infections, with an emphasis on self-assembled nanoparticles, will next be addressed in depth. Finally, the use of nanotherapeutics in the recent coronavirus epidemic will be presented.

## 2. Respiratory Viruses

Viruses are obligatory intracellular entities that are incapable of self-replication. It can guide the infected cell machinery to create additional virus particles. The genetic material of most viruses is either RNA or DNA, but not both at the same time. The nucleic acid molecules (DNA or RNA) may be single- or double-stranded (ssNA). The nucleic acid of an infectious virus particle (virion) is encased inside the capsid, made up of many replicates of a single or several distinct proteins. Some viruses, known as enveloped viruses, have a lipid envelope that is generated from the host cellular membrane during budding. Those viruses lacking this lipid membrane are called nonenveloped viruses. The range of hosts that a virus may infect is commonly used to categorize it. A virus that solely infects bacteria, for example, is known as a bacteriophage, or simply a phage. Several events highlight the lytic cycle of viral replication: adsorption, penetration, replication, and release of new virus particles. The outcome of these events is the death of the infected cell [7].

Only a small number of viral infections have clinical characteristics that may be used to determine the infection's aetiology. More often than not, generalized viral infections make the clinical picture less distinct, and as a result, a variety of viruses can cause illnesses with identical clinical symptoms, or syndromes.

The wide range of virus infections may include gastroenteritis and respiratory infections. Therefore, the diagnosis of virus infections depends on the infected cell types and organs showing the most virus concentration. Major concern was given to respiratory diseases, as they constitute a real health threat in the absence of effective antiviral drugs and vaccines.

Eight human respiratory viruses circulate commonly in all age groups: adenovirus (ADV), human bocavirus (HboV; parvovirus), human coronavirus (HcoV) and coronavirus related to the SARS-CoV (severe acute respiratory syndrome), human influenza virus (HIV), human metapneumovirus (HMPV; paramyxovirus), human parainfluenza virus (HPIV), human respiratory syncytial virus (HRSV), and human rhinovirus (HRV). Despite the fact that these respiratory viruses cause a large number of illnesses, there are now just a few preventative or therapeutic measures available.

In the next section, we highlight features of a number of viruses inducing common respiratory diseases.

### 2.1. Human Respiratory Syncytial Virus (HRSV)

The virus was originally identified as a cause of coryza in chimpanzees in 1956. It was soon discovered in children suffering from bronchiolitis and pneumonia. Because it produces multinuclear giant cell syncytia in tissue culture, it was named HRSV in 1957 [8, 9]. In 2016, HRSV was reclassified by the International Committee on Taxonomy of Viruses (ICTV), and since then the virus has been named human orthopneumovirus; however, we keep using the old name in the present review. HRSV is a ubiquitous virus and can infect very early in life. It is the most common viral pathogen causing severe lower respiratory tract infection (LRTI) and a primary cause of hospitalizations in young children, putting a significant strain on health care resources. Children less than 6 months account for nearly half of all LRTI hospital admissions and in-hospital fatalities caused by HRSV [10]. Presently, most people acquire HRSV infection before the second or third year of life, and reinfections are common throughout life. However, reinfection in older adults can occur causing upper respiratory tract infection and causing LRTI in 90% of the cases [11]. As a result, HRSV is a major cause of hospitalization, especially during the epidemic with both adults' and children's health being compromised. HRSV-associated hospitalization rates in adults approached those associated with influenza in 2015, with 3.4 million hospitalizations and 95,000–150,000 fatalities worldwide, and over 175,000 hospitalizations in children under the age of five in the United States per year [10, 12].

From a taxonomic point of view, HRSV is classified as a member of the *Paramyxoviridae* family [13], order of Mononegavirales, with a pleomorphic (80–350 nm in diameter) enveloped virus that is a member of the subfamily *Orthoparamyxovirinae* and is the type-species member of the *Orthopneumovirus* genus [14]. HRSV is an enclosed virus with a single-strand negative-sense RNA genome ((-) ssRNA) that is approximately 15 kb in size. The nucleocapsid protein (N), nucleocapsid-associated proteins (M2-1, P, and L), one M2-2 protein (the second ORF of the M2 gene), one matrix protein (M), three transmembrane proteins (F, G, and SH), and two nonstructural proteins (NS1 and NS2) are all encoded by the genome [15, 16].

**Figure 1:**
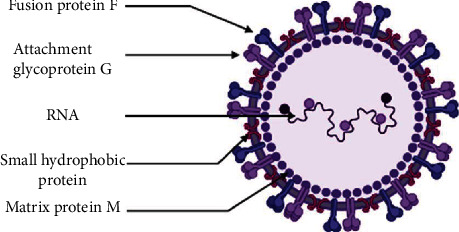
Schematic representation of HRSV.

The latter two proteins are expressed for IFN and apoptosis inhibition [16, 17] (Figure 1). The virus spreads between hosts via respiratory droplets or contaminated objects or surfaces. It infects the apical ciliated epithelial cells of the upper respiratory tract (URT) after 2 to 8 days of incubation in the nasopharyngeal or conjunctival mucosa [18]. Following cell attachment with the host cell membrane via G glycoprotein, the prefusion form of the HRSV-F glycoprotein links to nucleolin on the cell surface, causing membrane fusion and virus particle entry [19, 20]. The nucleocapsid is released shortly after that, including the viral genome and the N, P, M2-1, and L proteins [21–24]. The latter protein initiates the transcription and replication of the genome [25].

Finally, virus assembly takes place at the plasma membrane, where nucleocapsids bind to membrane viral glycoproteins that are found on the cell membrane [16]. The virus release occurs after clustering new mature virions at the apical surface [26].

Altogether, the virus replication in host cells may induce several complications, such as airway and alveolar obstructions, oedema, and pneumonia due to necrosis of respiratory epithelial cells, hypersecretion of mucus, and the accumulation of cellular debris [16, 18]. The immune response developed against HRSV is somehow impaired and does not induce long-term protection. The fact that most HRSV infections in healthy people are mild suggests that prior infection does produce significant immune protection [27]. The F and G proteins are the main antigenic targets of neutralizing antibodies around which vaccine and drug research have been putting the spotlight on. Palivizumab is a recombinant humanized monoclonal antibody and the only licensed prophylaxis treatment against HRSV, targeting the HRSV-F protein. However, HRSV resistance to palivizumab has been reported [28]. No vaccine has been approved yet. The hurdles in HRSV vaccine development include principally the immature immune system of neonates and the induction of low-affinity neutralizing antibodies [29].

### 2.2. Human Influenza Virus

The WHO estimates that more than 650,000 people die each year from flu-related respiratory illnesses throughout the world [30]. Since 2010, the CDC estimates that influenza has caused 9 million to 45 million illnesses, 140,000 to 810,000 hospitalizations, and 12,000 to 61,000 mortalities in the United States [31]. These numbers, often neglected by the public, expose influenza's weight on our population and our health care providers.

Country disease estimations play a crucial role in informing decisions about national influenza prevention and control programmes. Although reliable data about influenza disease are often missing from low- and middle-income countries. A, B, C, and D are the four types of influenza viruses known thus far. Seasonal outbreaks of illness are caused by human influenza A and B viruses every winter across the world, with type C viruses causing sporadic mild upper respiratory symptoms [32, 33]. Influenza D viruses are predominantly found in cattle and are not known to infect humans.

**Figure 2:**
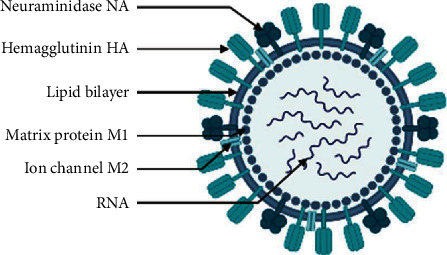
Schematic representation of an influenza virus.

The influenza A virus (IAV), on the other hand, causes the most serious clinical illness and is the most prevalent cause of seasonal epidemics and pandemics [34]. IAV strains are classified into subtypes based on two proteins found on the virus's surface: haemagglutinin (HA) and neuraminidase (NA). Both of these have a significant role in the development of disease. [35]. Influenza viruses are enveloped viruses of the family Orthomyxoviridae with a segmented ((-) ssRNA)) genome. The genome consists of eight segments [36, 37]. The termini of viral RNA connect with the viral RNA-dependent RNA polymerase (RdRp), which is made up of three protein subunits (PB1, PB2, and PA), whereas the rest of the viral RNA is bound by an oligomeric nucleoprotein (NP) [38–40]. At least 12 viral proteins are encoded by the genome, the majority of which are required for effective virus replication and virion production. The influenza virus genome is transcribed and replicated in the nucleus, unlike other ((-) ssRNA)) viruses. The M1 protein, which is located outside of the ribonucleoprotein complex and forms a layer underneath the lipid cell-derived envelope, is a significant structural component of the virus particles [41, 42] (Figure 2). The envelope of lipid cells is made up of three membrane proteins: HA, NA, and ion channel protein (M2). Fever, myalgia, headaches, malaise, sore throat, dry cough, and nasal congestion are all symptoms of influenza [43–45]. Gastrointestinal symptoms such as nausea, vomiting, and diarrhea are also frequent [46]. The incubation period for influenza is 1 to 4 days (from infection to onset of symptoms) [35]. Most people recover without medical intervention within a week after infection, although infection can sometimes lead to hospitalization and death, especially in those with underlying medical problems, infants/young children, and the elderly. Although the mechanism(s) underlying the RNA virus's evolution is (are) still not fully understood, the prevalence of new variants of IAV is driven by antigenic shift and drift phenomena [47].

The antigenic drift refers to the evolution of a virus as a result of changes in its genes that occur over time when the virus replicates. These mutations cause changes in the HA and NA surface proteins of the influenza virus, which are recognized by the immune system and capable of inducing an immunological response, including the development of antibodies that can prevent infection.

The impact of these mutations on the immune system's ability to identify novel variations is determined by the number of mutations acquired as well as their locations within surface proteins. The immune system may not be able to detect and prevent the newest influenza strains. As a result, a person is once again vulnerable to flu infection. To keep up with evolving influenza viruses, flu vaccine formulation must be examined and modified each year. In the spring of 2009, an H1N1 virus including genes from North American swine, Eurasian swine, humans, and birds emerged and swiftly spread, resulting in a pandemic [48, 49]. The WHO developed a Global Influenza Surveillance Network to track antigenic changes and provide yearly influenza vaccine composition recommendations [50]. Many vaccines are available in the market, but protection is down to 45% against 2019–2020 seasonal influenza A and B viruses according to the CDC [51]. Antiviral drugs and vaccines developed against the flu lack efficiency and broad spectrum coverage.

### 2.3. Human Coronaviruses (HCoVs)

Coronaviridae is a family that belongs to the Nidovirales order and the Coronavirineae suborder. The Coronaviridae family (CoVs) are subdivided into 4 genera: alpha, beta, gamma, and delta. Unlike alpha and beta CoVs, which only infect mammalian species, gamma and delta CoVs infect a wider range of animals, including birds. CoV infections in humans and animals mostly cause respiratory and gastrointestinal illnesses [52, 53].

**Figure 3:**
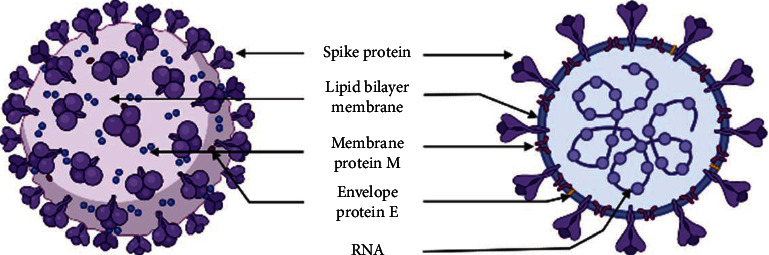
Schematic representation of CoVs.

They are enveloped positive-sense single-stranded RNA viruses ((+)ssRNA), with club-like spikes protruding from their surface, a huge RNA genome, and a unique replication mechanism. They may induce a wide range of illnesses in animals, including enteritis in cows and pigs, upper respiratory disease in chickens, and fatal respiratory infections in human. For a long time, CoVs were not thought to be particularly harmful to humans, as demonstrated by HcoV-229E, HcoV-OC43, HcoV-NL63, and HcoV-HKU1. They have been recognized for a long time to cause seasonal, generally minor respiratory tract infections. However, the severe acute respiratory syndrome (SARS) outbreaks in Guangdong Province, China, in 2002 and 2003 [54–57] followed by the Middle East respiratory syndrome CoV (MERS-CoV) in Middle Eastern countries [58], have demonstrated that CoVs may cause more serious respiratory infections in humans. SARS-CoV and MERS-CoV were reported to be directly transmitted to humans by market civets and dromedary camels, respectively. Both viruses, however, are believed to have originated in bats [59, 60]. The emergence of a new SARS, caused by a newly emerged human CoV strain in December 2019 in China [61, 62], named SARS-CoV-2, has further shown the unexpected severe character of these CoVs. The disease quickly became a pandemic and constituted a global threat for both human health and world economic trade. With 185 million confirmed COVID-19 cases, including 4 million deaths, reported by July 2021, the illness soon became a pandemic, posing a global danger to human health and global economic commerce [63]. The replicase gene, which encodes nonstructural proteins (NSPs), takes up two-thirds of the genome (approximately 20 kb), whereas structural and accessory proteins take up only around 10 kb. The spike (S), membrane (M), envelope (E), and nucleocapsid (N) proteins are coded by the 3′ end of the viral genome (Figure 3). The angiotensin-converting enzyme 2 (ACE 2) receptor binds to the spike protein, which allows SARS-CoV and SARS-CoV-2 to infect cells [64, 65].

The ACE 2 receptor is not only present in epithelia of the lung but also, with different expression rates, in oral and nasal mucosa, nasopharynx, stomach, small intestine, colon, skin, lymph nodes, arterial and venous endothelial cells, thymus, bone marrow, spleen, liver, kidney, and brain which could explain the pathogenesis encountered following viral infection [66]. CoVs are widely spread through respiratory droplets. Infection can occur following direct exposure and inhalation of droplets or indirect contact with nasal, conjunctival, or oral mucosa [64]. Sia and collaborators (2020) described the pathogenesis and transmission of SARS-CoV-2 in golden hamsters, a perfect animal model with ACE 2 receptors able to support SARS-CoV and SARS-CoV-2 virus replication [67, 68]. They proved the presence of SARS-CoV-2 viral antigens in nasal mucosa, and bronchial epithelial 2 to 5 days after inoculation. The study showed replication of the virus in the upper respiratory tract pursued by lower infection within the lungs with a strong innate immune response which could explain the pulmonary symptoms like those of SARS and MERS, i.e., fever, dry cough, pharyngitis, shortness of breath, joint pain, and tiredness in infected patients [67–69]. IgA, IgM, and IgG antibodies were detected after the symptomatic onset, indicating a B-cell-mediated humoral immune response against the nucleocapsid protein N and the spike protein S. Viral clearance is dependent on the T-cell immune response to suppress infected cells and stop viral replication [69]. Protective immunity duration post-SARS-CoV-2 infection is an uncertain point and subject of debates. However, studies suggest that immunological memory could last 3–8 months [70, 71]. All treatments used are not specific for SARS-CoV-2, but they tend to lower the complication risk, enhance the patient's overall comfort, and decrease his hospital stay. Fortunately, more than 50 vaccine candidates are currently in trials [72] with more than 10 approved worldwide, including BioNTech/Pfizer BNT162b2, Moderna mRNA-1273, Gamaleya Sputnik V, Oxford/AstraZeneca AZD1222, Sinopharm BBIBP-CorV, Sinovac CoronaVac, Sinopharm Inactivated, FBRI EpiVacCorona, and CanSinoc Ad5-nCoV [34, 73]. Among these, both Moderna and BioNTech mRNA encoding for the spike protein are encapsulated with lipid nanoparticles to protect the mRNA from degradation by nucleases and provide its cell internalization [74, 75], while Gamaleya Sputnik V, Oxford/AstraZeneca, and CanSinoc vaccines are based on adenoviruses as vectors with E1 and E3 deletions encoding for the full-length S protein [74]. Sinopharm and Sinovac vaccines are inactivated SARS-CoV-2. The world's leading vaccine approved in 55 countries is BioNTech/Pfizer's LNP vaccine being the first ever RNA-based vaccine, licensed for human use with more than 90% efficacy [76]. This approval brings to light the open future of nanotechnology in the field of vaccination.

### 2.4. Human Adenoviruses (HAdVs)

**Figure 4:**
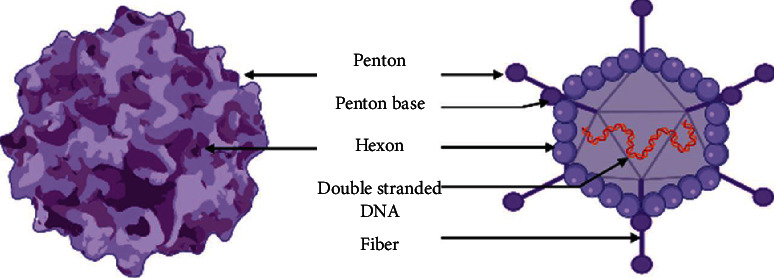
Schematic representation of adenovirus.

Human adenovirus (HAdV) infections represent 5 to 10% of pediatric and 1 to 7% of adult respiratory tract infections (RTI) [77]. HAdVs are nonenveloped double-stranded linear DNA viruses coated with a 70–150 nm sized icosahedral nucleocapsid. This latter is made of 3 different capsomeres:hexons, penton bases, and penton fibers through which the virus binds to the host cell's Coxsackie B and adenovirus receptor (CAR). The viral entry is a receptor-mediated endocytosis assisted by cell surface integrins. Acidity within the endocyte cleaves the fibers and exposes pentons that promote endosomal membrane lysis and capsid release in the cytoplasm [78] (Figure 4). Thereafter, the viral capsid reaches the nucleus and attaches to the nuclear pore complex (NPC) via an interaction with the hexon. The viral genome is then injected into the nucleus to proceed to early and late replication phases [78]. Fifty-one human adenovirus serotypes have been identified and distributed in six species, A–F.

Respiratory disease, gastroenteritis, and keratoconjunctivitis are the clinical diseases expressed by adenoviruses depending on their cell tropism. Among the HAdV-associated respiratory diseases, serotypes 1–7, 11, 14, 16, 21, and 50, are considered to be the main pathogens that cause respiratory tract infection [79]. Infections are more frequent in young children because of the lack of humoral immunity. Epidemics can occur in healthy adults, particularly military recruits, or children causing an URT infection (mild colds) in most cases, while severe cases appear mostly in immunocompromised patients with LRTI. Untreated HAdV can lead to viral dissemination and high mortality [77, 78]. Treatment is symptomatic and antiviral therapy using ribavirin trifluridine and cidofovir for severe AdV infections has been reported. Live oral vaccines against AdV types 4 and 7 are very effective in preventing respiratory infection and are routinely used by United States soldiers, but not yet available to civilians [77]. Since AdVs readily infect humans, they have been used as gene therapy vectors and vaccine delivery systems. In the COVID-19 pandemic, the Oxford/AstraZeneca team worked with a modified version of a chimpanzee adenovirus, known as ChAdOx1. It can be introduced into cells, but it cannot replicate inside them [79]. The Sputnik V vaccine is based on a heterologous recombinant adenovirus approach using adenovirus 26 (Ad26) and adenovirus 5 (Ad5) as vectors for SARS-CoV-2 spike glycoprotein expression [80].

## 3. Classical Antivirals and Vaccines

### 3.1. Anti-RSV

RSV treatment is mainly supportive. Maintaining hydration and oxygenation within the physiological norms is the basis of RSV management. Nevertheless, bronchodilators, corticotherapy, decongestants, and antiviral agents have been tried in multiple studies with no significant impact on the course of the illness [81]. Ribavirin (a nucleoside analogue) is approved for the treatment of patients with severe RSV infection only [82]. Its efficacy has not been proven, and there is no sufficient evidence to confidently state whether or not it is clinically effective in mild to moderate RSV infection. Therefore, its routine use is not recommended in RSV patients, and only severe RSV for LRTI should be treated with ribavirin [81, 83]. Prevention could be established with palivizumab, a monoclonal antibody (mAb) directed against the RSV fusion (F) protein [81]. However, a double-blind randomized clinical trial on over 400 infants did not provide significant effectiveness [84]. The high cost of both antiviral treatment ($1,192 to $2,085 a month) [85] and antibody prevention ($1,866 per vial in the US) [86] raised ongoing cost-effectiveness controversies. Failing to meet sufficient effectiveness with these compounds, researchers are more oriented to vaccine development, even though several antiviral agents such as enzaplatovir, presatovir, and caplacizumab are currently under investigation [87]. Vaccination against RSV remains on hold as there is no licensed vaccine available so far. In addition, RSV vaccine development is very complicated due to antigenic diversity, the immunization of young infants who may respond inadequately to vaccination, and the history of the formalin-inactivated RSV vaccine. The latter was used in the early 1960s; it not only failed to protect, but also generated an exaggerated clinical response in infants [88]. Despite the immense effort by researchers putting up many types of vaccines in clinical trials [89], no conventional vaccine was potent enough to get licensed.

### 3.2. Anti-Influenza

Although influenza viruses cause mild illness with quick recovery in healthy adults, elderly and immunocompromised patients are often exposed to clinical complications needing medical care. The main part of treatment in influenza patients is supportive therapy with fever and hypoxemia management, although the Guidelines by the Infectious Diseases Society of America (IDSA) recommend antiviral treatment for any patient with suspected or confirmed influenza who is hospitalized; has severe, complicated, or progressive illness; or is at a higher risk for influenza complications [90]. Several antiviral medications are recommended for treatment and chemoprophylaxis of influenza that depends on the evolution of symptoms and age of patients: oral oseltamivir (influenza A and B), inhaled zanamivir (influenza A and B), intravenous peramivir (influenza A and B), and oral baloxavir (influenza A and B). There are some restrictions in their use, and they will be discussed below. Oral oseltamivir Tamiflu® (neuraminidase inhibitor (NAI)) is the gold standard antiviral in these three circumstances. With proven efficacy, it is prescribed for both treatment and prophylaxis of influenza in adults and children older than one year old. In 2000, Treanor and collaborators (2000). demonstrated a reduced illness duration and severity by more than 30% and 35%, respectively [91]. In addition, the highly protective efficacy of oseltamivir was shown when used as postexposure prophylaxis in households. More than 80% of laboratory-confirmed influenza cases were prevented with oral oseltamivir prophylaxis treatment in households [92]. Following its oral administration, the dose is widely distributed in the body and excreted as oseltamivir carboxylate (the active form) mainly through the kidneys with a short 1–3 hours half-life [93]. Zanamivir Relenza® (NAI) commercialized as an oral inhaled powder is also an alternative for influenza A and B treatment or prophylaxis. Monto et al. showed through a randomized clinical trial that zanamivir was 67% efficacious in preventing laboratory-confirmed clinical influenza cases [94]. Both antiviral compounds are most effective when treatment is initiated within 48 hours of the onset of symptoms. Other antiviral agents such as intravenous peramivir or oral baloxavir may be used for treatment [95]. Besides oseltamivir and zanamivir, amantadine and its analogue rimantadine represent the first antivirals licensed against influenza. They act as replication blockers through their interaction with the viral M2 protein. Thus, they inhibit viral uncoating and entry into the cell. Both of these agents are only effective against influenza A infections with high rates of resistance exceeding 30% and up to 80% [81], which makes these drugs useless in the treatment of influenza infection [96].

Antiviral therapy with neuraminidase inhibitors is associated with several side effects, such as bronchospasm and reduction in airflow related to zanamivir and nausea, vomiting, and abdominal pain related to oseltamivir in 10% of treated patients [97]. Flu vaccination is available with different types of vaccines in the market. The CDC recommends the use of any licensed vaccine including inactivated influenza vaccine (Fluzone®), recombinant influenza vaccine (Flublok®), or live attenuated influenza vaccine (FluMist®) [98]. All the abovementioned vaccines offer protection against the selected strains by WHO. In fact, every two years, WHO analyses surveillance data and laboratory and clinical studies to determine the circulating flu viruses that should be included in the vaccine [99]. The conventional vaccine approach comes with many limits, such as virus reactivation regarding live attenuated vaccine, lack of immune response to recombinant vaccine needing the addition of a potent adjuvant, and the administration of multiple dosages. Moreover, the short length of protection is the setback of a killed vaccine.

### 3.3. Anti-SARS-CoV

There is no specific anti-SARS treatment so far, but many potential antiviral molecules have been investigated *in vitro* and in patients. Following the SARS-CoV 2003 outbreak, ribavirin (RBV) has been used at therapeutic doses as a standard anti-SARS agent in Hong Kong, Canada, and other countries. However, in vitro and in vivo studies indicated that the virus was modestly sensitive to ribavirin at high doses [77]. Furthermore, ribavirin showed side effects including haemolytic anaemia in 33% to 73%, hypocalcaemia in 58%, and hypomagnesaemia in 46% of patients [100]. Besides, clinical studies evaluating the efficacy of ribavirin against SARS-CoV were inconclusive. Confronting the new SARS pandemic, a recent retrospective cohort study including 2,037 patients with COVID-19 concluded that RBV/IFN-*α* therapy did not show any benefit in improving patient outcomes [101]. Many other antiviral compounds, such as interferons, were used in the hope of efficiently managing the 2003 and 2019 outbreaks. Protease inhibitors have been used in Hong Kong with promising result. Ritonavir and lopinavir association (Kaletra), used in SARS-CoV patients, was associated with lower death rates and intubation rates and a significantly lower incidence of ARDS [100]. Otherwise, ritonavir and lopinavir (LPVr) used in clinical trials with COVID-19 patients reported possible effectiveness with reduced mortality at 28 days and shortened ICU admissions and time to discharge. The conducted studies are limited methodologically and quantitatively, as they lack a respectful sample size. More sophisticated and larger studies need to be further conducted to determine the efficacy and safety of LPVr for COVID-19 [102]. On the other hand, remdesivir is the only antiviral agent approved by the FDA for COVID-19 patients that was recommended by the Infectious Diseases Society of America (IDSA) to be used for hospitalized patients with severe COVID-19, defined as patients with SpO_2_ ≤ 94%, or patients who require supplemental oxygen, mechanical ventilation, or extracorporeal mechanical oxygenation [103]. Due to the huge urge to control 2019s devastating pandemic, the biomedical community conducted numerous clinical trials on previously known antiviral agents for the treatment of COVID-19, i.e., remdesivir. Some studies proclaim that remdesivir was potent against SARS-CoV-2, exhibiting a shorter time to recovery, better clinical status, and less mortality [104, 105]. However, WHO concluded that it had little or no effect on patients [106, 107]. The drug continues to be endorsed by the FDA and EMA in critical conditions, but further studies need to be undertaken to prove its efficacy.

Vaccination against SARS-CoV-2 is one of the most outstanding achievements in human medical history. One year after the virus discovery, the FDA issued the first emergency use authorization for the Pfizer/BioNTech COVID-19 vaccine [73]. This was the first mRNA and nanoparticle vaccine to be approved for human use. Soon after, many others got licensed, such as the Moderna mRNA-1273 vaccine, the Oxford/AstraZeneca AZD1222, the Janssen (Johnson & Johnson) Ad26.COV2.S, and the Sinovac CoronaVac [73]. However, China led the way to the first approved and effective vaccine in June 2020, but only for limited use in the Chinese military. It is a DNA vaccine coding for the spike protein incorporated in a nonreplicating adenovirus as a delivery system to human cells [73]. The second in the race was Russia with approval of its Sputnik V vaccine for widespread emergency use in August 2020 [73].

### 3.4. Antiadenovirus

There is no clinically approved antiviral agent against AdV infections; however, multiple drugs have been tested, such as ribavirin [108, 109] and cidofovir [110].

In 2011, the adenovirus vaccine was used by the US military to reduce acute respiratory disease. The vaccine is an oral live virus type 4 and type 7 produced by Teva Pharmaceutical Industries Ltd. under contract to the U.S. Army. There is currently no adenovirus vaccine available to the general public [111]. However, adenovirus is a common vaccine vector under promising clinical trials for other pathogens such as tuberculosis, Ebola, Zika, HIV, and SARS-CoV-2 [73, 112]. The 2020s pandemic represented the right set of circumstances to give the green light to several adenovirus-based vaccines against SARS-CoV-2 such as Sputnik V, Janssen Ad26.COV2.S, Oxford/AstraZeneca AZD1222, and CanSino Ad5-nCoV [73].

## 4. Nanoparticles as a Vaccine/Drug Delivery System

Introduced in 1959 by the physicist Richard Feynman, nanotechnology is now an integral part of science. Its use in medicine came with an outstanding breakthrough in delivery systems. With the possibility to control size, shape, and compounds, nanoparticles (NPs) offer an advanced ground for the diagnosis and treatment of diseases. Furthermore, NPs can carry drugs either by encapsulation or by conjugation with the possibility of active or passive targeting. With such rigorous control, promising pharmacokinetics can be achieved, such as prolonged drug half-life, enhanced drug efficacy, and decreased toxicity [113]. Many nanobased drugs and nanodelivery systems are FDA and EMA approved for human use, and more similar drugs are being investigated in clinical trials [114]. NPs can be classified into several types, according to size, morphology, and physical and chemical properties. They are generally classified into organic (dendrimers, micelles, liposomes, ferritin, etc.) and inorganic particles (iron oxide nanoparticles, silver or gold nanoparticles, carbon dots, etc.) [115].

### 4.1. Liposomes

**Figure 5:**
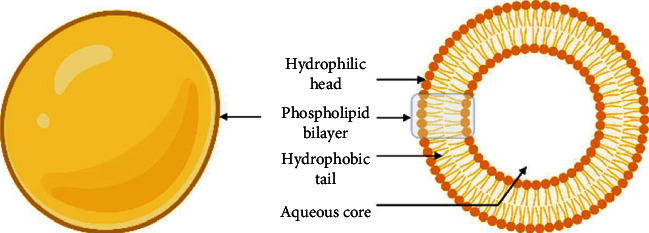
Schematic representation of liposomal drug delivery systems.

Liposomes were first described by Alec Bangham, in 1961. Since then, they have been investigated as plausible drug delivery systems [116]. They are spherical nanovesicles, consisting of one or more lipid bilayers. These layers can either be composed of natural or synthetic phospholipids, usually phosphatidylcholine [117] (Figure 5). The liposome size ranges between 30 and 1000 nm, depending on the preparation method. This unique structure allows loading hydrophilic drugs in the aqueous core, as well as hydrophobic therapies within the acyl hydrocarbon chains of the lipid bilayers [118]. On the basis of their size and number of bilayers, liposomes can be classified into multilamellar vesicles (MLV, with several lamellar phase lipid bilayers), small unilamellar liposome vesicles (SUV, with one lipid bilayer), and large unilamellar vesicles (LUV) [117]. Their production is a simple procedure, consisting of three main steps: lipid drying, lipid hydration, and purification.

Nanoformulations of existing drugs with low bioavailability or high toxicity have benefitted from the stability and improved biodistribution that liposomes provide. In this context, PEGylated liposomes (with a hydrophilic polymer chain) were developed to overcome short half-life and instability due to hydrophobic interactions with opsonins and rapid uptake via the mononuclear phagocytic system [119]. The main example of this achievement is Doxil, a PEGylated liposomal formulation of doxorubicin that was indicated for the treatment of patients with ovarian cancer, for whom disease has progressed or recurred after platinum-based chemotherapy. Liposomes were the first nanostructures to get Investigational New Drug (IND) status by the FDA [120]. Soon after, Doxil was approved in 1995 for better cardiac safety, fewer side effects, and a 16-fold enhancement of drug levels in malignancies when compared to free doxorubicin [121]. Since its approval, Doxil has been widely used [122, 123] and liposomes are now the leading nanostructure, as an approved delivery systems. Further liposome nanoformulations were licensed, such as AmBIsome and Abelcet for amphotericin with reduced nephrotoxicity and Vyxeos for daunorubicin and cytarabine codelivery. Many others are still under clinical investigation, such as Arikayce, an inhaled liposomal formulation of amikacin for the treatment of serious chronic lung infections [120].

Many immunoliposomes are also under promising clinical investigations. These liposomes are endowed with surface antibodies or ligands for targeted site drug delivery, reducing side effects and toxicity [124]. Liposomes are also an interesting antigen carrier for vaccines as they can carry viral proteins, DNA, and RNA. Moreover, they are capable of inducing cellular or humoral immune response depending on their charge, size, and lipid composition [125]. For instance, unsaturated lipids were shown to induce Th2 responses, whereas saturated lipids promote Th1 immune response [126]. While small-sized liposomes have been shown to be uptaken by DCs, larger ones are phagocytosed by macrophages. Moreover, small liposomes (100 nm) would mostly induce Th2-dependent responses, while larger liposomes (400–1000 nm) would stimulate Th1-based responses [126]. Since their first application as a vaccine delivery system in 1974, two liposome-based vaccines have been approved for human use: Inflexal V for influenza and Epaxal for hepatitis A [127].

For DNA- and mRNA-based vaccines, the lipid composition should be adjusted to provide tight interactions with the negative charges of nucleic acids. As the number of positively charged phospholipids is scarce, synthetic lipids are usually designed and auto-assembled similarly to liposomes, leading to the so-called lipid nanoparticles (LNP). Cationic liposomes or LNPs not only protect DNA and RNA from degradation but are often associated with a greater immune response due to favorable electrostatic interactions with anionic cell membranes. Nevertheless, positively charged carriers also suffer from rapid elimination from the bloodstream upon systemic injection, which is why ionisable lipids have been proposed [128]. These lipids, positively charged at low pH and neutral at physiological pH, provide both nucleic acid interaction and long-circulation time. Associated with exchangeable lipid-PEG to provide nucleic acid release after cell internalization, these liposomes are a condensed state of innovation. In parallel, amazing discoveries have been made in the field of RNAs. The concomitant discovery of the role of siRNA or mRNA and lipidic autoassembled systems led to the amazing rapidity to conceive a molecule encoding for the spike of SARS-CoV-2. The simultaneous progress made in the last thirty years on RNAs and liposomes dedicated to nucleic acid delivery finally led to an efficient delivery system for the first siRNA approved drug, which reached the market in 2018 [129]. This overall success led to the rapid design of the actual mRNA delivery systems in the context of SARS-CoV-2. Thanks to these advances, RNA liposomal vaccines against SARS-CoV-2 have been approved by the FDA and EMA less than a year after the worldwide diffusion of the virus. The Pfizer and BioNTech vaccine made with mRNA and lipidic nanoparticles showed 90% efficacy on 3000 patients, and the Moderna LNP RNA vaccine reached a 94.1% effectiveness (CDC). Little is known concerning the mRNA-lipid structure, even though some information is now reported [130]. Nevertheless, this efficacy and the easy design of mRNA opens the way for future lipid-based drug delivery systems [131]. Many other lipid-based vaccines against SARS-CoV-2 and influenza are still under investigation (Table 1). This sophisticated nanocarrier offers broad flexibility in drug or vaccine delivery with better pharmacokinetics such as higher stability concerning fragile molecules or proteins as discussed earlier, sustained release of cargo, lower therapeutic doses, and reduced toxicity of the encapsulated agent. Moreover, liposomes can play a key role in vaccination as some lipid compounds have adjuvant properties. Although the DDS is known to be moderately immunogenic or nonimmunogenic, it can be rendered immunogenic by modifying its surface [132]. The main liposome drawbacks concern cationic lipids, which exhibit toxicity and low intracellular release of their payload. However, ionisable lipids partially improve these side effects. From charged to uncharged, the downside is their high cost of production, which limited their ordinary usage till the COVID-19 crisis. Now that the production process and site are up and running, it offers a broader perspective on lipid-based carriers.

### 4.2. Polymeric Nanoparticles

**Figure 6:**
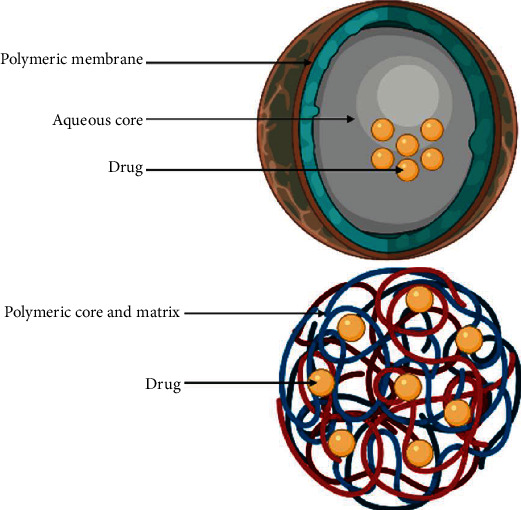
The two main types of polymeric nanoparticles known as the nanosphere (matrix system) and the nanocapsule (reservoir system) with different drug-loading modalities.

Polymeric nanoparticles (PNPs) are one of the most investigated NPs. Endowed with biocompatibility and meticulous control over shape, size, and components, they offer a magnificent drug/vaccine delivery system. PNPs are made from natural polymers (chitosan, albumin, alginate, and heparin) or synthetic polymers ((N-(2-hydroxypropyl) methacrylamide, copolymer, polyethylene glycol (PEG), and poly(lactic acid)/poly(lactic-co-glycolic acid)), as their name suggests. They are utilized in a number of biological applications [130]. There are two forms of polymeric NPs: nanospheres and nanocapsules. The nanospheres have drugs adsorbed on their surface or incorporated in the matrix, whereas nanocapsules have drugs enclosed in their core (Figure 6). Polymer NPs may be made in a variety of shapes and sizes, ranging from 10 nm to 1000 nm [120]. Some polymeric NPs can help with drug release for weeks without accumulating in the body; they can also help with regulated release, improved cellular uptake, drug molecule protection from degradation, site-specific delivery, minimal toxicity, and theranostic properties [118]. Therefore, polymeric NPs are being explored as potential carriers for a variety of medicines, including cancer therapies, cardiovascular disease treatments, diabetes treatments, and vaccines [118]. The predominance of polymers among authorized and currently studied nanodrugs demonstrates their value in enhancing traditional diagnostic and therapeutic medicine (indicated in Table 1).

#### 4.2.1. PLGA Nanoparticles

Poly(lactic-co-glycolic acid) (PLGA) NPs are the most widely used PNPs, since their FDA and EMA approval. PLGA is a synthetic polymer, consisting of linked monomers of lactic acid and glycolic acid. Both of these elements are biodegradable, biocompatible, and efficiently processed by the body via the Krebs cycle after hydrolysis of the polymer [133]. Before any processing, NPs, circulating within the bloodstream encounter opsonins. Opsonized NPs get internalized via the RES, which leads them to the liver or the spleen. This system exhibits a substantial biological barrier to NP distribution and bioavailability. To address these limitations, several modified surface PNPs have been studied. Much like liposomes, PEGylation of polymeric NPs is a key to bypass the RES. Grafting hydrophilic groups, such as polyethylene glycol (PEG), hide the hydrophobic surface and limit recognition by the immune system. Moreover, they inhibit hydrophobic and electrostatic interactions with plasma opsonins, giving longer drug half-life and better distribution [134]. Using this technology, interferon gamma beta-1a is now administered once every two to four weeks, instead of daily administration for the treatment of relapsing forms of multiple sclerosis thanks to a licensed PEGylated formula named Plegridy® [120]. In addition, ligands and antibodies can be grafted on the polymeric surface, giving the system further affinity to targets. PLGA-based NPs can protect antigens from degradation for four weeks, which is very helpful when it comes to mucosal vaccination [133]. Moreover, PLGA NPs promote antigen internalization by APCs and facilitate antigen processing and presentation to naïve lymphocytes [134]. The most common technique used for the preparation of PLGA nanoparticles is the emulsification-solvent evaporation technique that allows the encapsulation of hydrophobic drugs. Other techniques, such as the double emulsion W/O/W, were used to encapsulate hydrophilic drugs, like peptides, proteins, and nucleic acids. PLGA nanoparticles can be developed by the nanoprecipitation method, also called the interfacial deposition method [134]. Other techniques exist, such as the spray-drying method [134]. Drug loading into NPs is achieved by two methods: whether during the NP production or the adsorption of the drug on NPs after their production. This type of drug delivery system (DDS) comes with controlled release of loaded substances, going from hours to several months. The two major mechanisms associated with drug release from PLGA-based DDSs are diffusion and degradation/erosion [135]. Astonishing therapy outcomes associated with PLGA nanoparticles were achieved, with greater cargo stability, longer circulation time, longer half-life, and extended release. The polymeric nanoparticle, Capoxone® (glatiramer acetate), approved by the FDA in 1996, is indicated for the treatment of multiple sclerosis symptoms, acting as an immunomodulator with greater circulation and stability [120]. Multiple other polymeric nanoparticles were licensed, such as Neulasta® (Pegfilgrastim), for the treatment of chemotherapy-induced neutropenia, and Adynovate (antihemophilic factor) and Rebinyn (coagulation factor IX), for the treatment of acute bleeding in hemophilia A and B, respectively [120]. As far as we know, no antiviral drug with polymeric formulation has been approved; however, nanoformulations of Efavirenz and Lopinavir have demonstrated effective suppression of HIV in preclinical studies [136]. Considered as a pivotal DDS, PLGA nanoparticles have a recognized position in drug and vaccine delivery. This position stands behind different and numerous clinical trials and studies cited in Table 1. Influenza, parainfluenza, and respiratory syncytial viruses were the center of attention in PLGA nanoparticle-based vaccines.

Polymeric delivery systems come with great assets such as EMA and FDA approval for human use. Their biodegradability and biocompatibility, therefore, have low toxicity. Besides, with PLGA NPs, we can secure sustained release and targeted delivery of cargo to specific organs or cells [137]. Finally, the production methods and formulations are well described in the literature. However, PLGA NPs have low drug loading even with their high encapsulation efficiency. Compared with liposomes and their sustained release, these polymeric NPs usually offer high burst release of cargo. This latter cannot be sensitive to low pH (such as RNA or DNA) due to acid formation during PLGA breakdown. Furthermore, the scale-up and high cost of these systems can be roadblocks for their development [137]

#### 4.2.2. Chitosan Nanoparticles

Chitosans (CS), biopolymeric nanoparticles, are made of the second most abundant natural polysaccharide, chitin. This sugar is made up of a mixture of -(1-4)-linked-D-glucosamine and N-acetyl-D-glucosamine, as well as N-(2-hydroxypropyl) methacrylamide/N-isopropylacrylamide, which may be found in crustacean shells like shrimp or crab shells and fungus cell walls. First, it was discovered by French Professor Henri Braconnot in 1811. Since then, continuous research and application trials have been conducted within the pharmaceutical field due to its attractive features, to finally reach approval by the US FDA for tissue engineering and drug delivery [138]. It is a cationic, highly basic and biocompatible polymer with low toxicity and immunogenicity. CS has been reported to enhance drug bioavailability for both oral and nasal routes due to its mucoadhesive property and the positive surface charge, allowing interaction with the cell's negative membrane.

Emulsion cross-linking, emulsion-droplet coalescence, ionic gelation, reverse micellar technique, and a chemically modified chitosan method are five ways for its manufacture. Ionic gelation, on the other hand, is the most frequent technique for making chitosan NPs. Sodium tripolyphosphate (TPP) is used to cross-link CS precursors, resulting in large-sized (100–300 nm) polydisperse particles [139].

A pitfall associated with CS is its restrained spectrum of drug loading. Indeed, CS NPs are only able to load hydrophilic drugs like various antiviral molecules, proteins, and RNAs. The incorporation of drugs may be performed either during nanoformation or by incubating preformed nanoparticles in the drug solution [140]. Following administration, CS tend to release their cargo by three main mechanisms: desorption, diffusion, and polymer erosion release. [140].

Although CS has been studied for various administration routes, the mucosal delivery is considered the suitable one, due to the mucoadhesive signature. On the other hand, CS nanoformulations have poor stability, making it a challenge to produce shelf-stable NPs [140]. CS derivatives have been developed in order to improve permeability and mucoadhesive property. Also, hydrophobic drugs for chemotherapy can be incorporated with these modified amphiphilic CS [141]. This form of NPs could be a suitable way to deliver antiviral drugs, characterized by their high hydrophilicity and thus very low per os bioavailability.

Chitosan vaccines containing influenza, diphtheria, and pertussis antigens for nasal delivery were prepared by Illum et al. In systemic vaccine delivery, chitosan acts as an adjuvant. Activation of macrophages occurs after the uptake of chitosan. Chitosan has been widely used for DNA mucosal vaccines. The same authors have developed a chitosan-based DNA flu vaccine along with other chitosan antigen-based vaccines still in clinical trials [142], as summarized in Table 1.

As discussed earlier, chitosan proposes low toxicity with mucoadhesive properties, making it appropriate for mucosal routes. However, this DDS lacks sufficient stability, making it a hurdle for industrial high-scale production. It is only appropriate for the encapsulation of some hydrophilic drugs such as proteins and RNA, but its low immunogenicity may be unsolicited [140]. All these pitfalls limit researchers' orientation to chitosans as a DDS.

#### 4.2.3. Dendrimers

**Figure 7:**
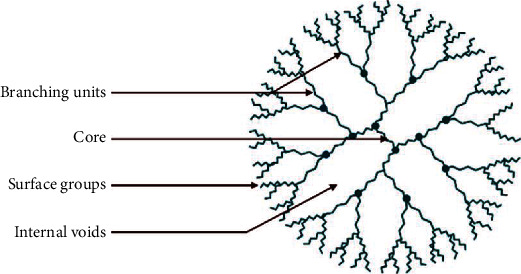
Schematic representations of dendrimers.

Dendrimers are polymeric nanosized particles, made of a central polymer or a molecule from which emanates symmetrical branches forming 3D hyperbranched monodisperse structures (Figure 7). These well-defined homogeneous structures are made of three units: a central core, an inner shell, and an outer shell containing numerous functionalities [182, 183]. Their unique architecture makes them excellent candidates as drug or vaccine delivery agents. In fact, substances can be either entrapped in the inner shell voids or carried on their surface via electrostatic interactions or chemical conjugation [118]. Moreover, these tree-like structures offer a broad spectrum of assets, such as controlled size, weight, and drug release, low toxicity, high loading capacity, targeted delivery, and excellent cell uptake [118, 183].

Dendrimers can be attained whether through a divergent method or a convergent approach. In these two techniques, monomers are added sequentially by click chemistry or lego chemistry to the initiator [118, 183]. Meticulous control of the dendrimer's pharmacological and physicochemical characteristics is conducted by using different polymers, monomers, and functional groups [118, 182, 183]. Several kinds of dendrimers have been reported, such as polyamidoamine (PAMAM) dendrimers, polypropylene imine (PPI) dendrimers, liquid crystalline dendrimers, peptide dendrimers, glycodendrimers, and poly-L-lysine dendrimers (PLL). The latter, functionalized with naphthalene disulfonate groups, is the only approved dendrimer for the prevention of bacterial vaginosis, known as VivaGel® [118]. The approval concerns the EU region and Australia, while FDA authorization is still pending. PAMAM, PLL, and carbosilane dendrimers have been studied as antiviral NPs against influenza via sialic acid functionalization. Sialic acid linked to PAMAM dendrimers completely protected against infection in a murine influenza pneumonitis model [184]. A recent study, using PAMAM dendrimers as an influenza H5-DNA vaccine delivery system, evokes successful protection against the H5N1 virus [185]. Having such a low molecular weight, dendrimers are weakly immunogenic to nonimmunogenic when it comes to vaccine delivery. Protein conjugation, which implies higher molecular weight and multifunctional immunogens, could overcome this problem [186]. In this regard, multiple antigenic peptide (MAP) dendrimers were introduced as VDS with the ability to present multiple copies of an antigen or multiple antigens to the immune system, simultaneously [186]. Dendrimers are expensive DDS with high costs for their synthesis. Moreover, their surface-group nature/generation-dependent cytotoxicity, which can be significant with cationic dendrimers, constitutes a hurdle for their clinical application. Their tunable chemical and physical properties, cargo solubility, increase of lipophilic drugs, and possible targeted delivery or multiantigenic vaccine presentation, on the other hand, impose them back as a significant DDS and encourage the pursuit of new studies [187].

### 4.3. Micelles

**Figure 8:**
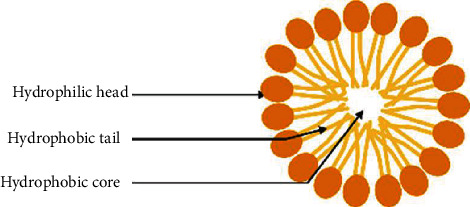
Schematic representations of micelle.

The concept of “micelles” was introduced to describe self-assembling structures with particle diameters from 5 to 100 nm range [118]. The amphiphilic molecules aggregate at a certain temperature (critical micelle temperature (CMT)) and a well-determined concentration (critical micelle concentration (CMC)) [118]. The core of the micelles is formed by the hydrophobic fragments, carrying poorly water-soluble drugs (Figure 8), while the hydrophilic surface can be conjugated to the hydrophilic active component and then allow higher drug loading and minimal premature release [188].

Additionally, targeted drug delivery is a promising approach with ligand or antibody surface grafting. Various multifunctional micellar formulations were elegantly designed and are under active investigations [189]. Endowed by greater stability *in vivo*, polymeric micelles (PMs) hold enhanced qualities compared to conventional micelles. PMs are made of amphiphilic polymers and, most likely, the FDA-approved PEG. Other polymers have been utilized, such as poly(N-isopropylacrylamide), poly(methacrylic acid), and biodegradable poly(esters), which includes poly(glycolic acid), poly(D-lactic acid), poly(D,L-lactic acid), copolymers of lactide/glycolide, and poly(*ε*-caprolactone) [190]. Various techniques like solvent extraction, dialysis, and solution casting methods are used for formulating polymeric micelles, and with the appropriate components, tailored structures and characteristics of micellar NPs can be achieved, offering optimum drug loading and release. Estrasorb, a promising DDS with a FDA-approved formulation (oestradiol hemihydrate, Novavax, Inc.), is indicated for moderate-to-severe vasomotor symptoms associated with menopause. This micellar formulation led to stable levels of oestradiol for eight to 14 days. More are under clinical investigation, and most of them are cancer therapeutics such as cisplatin and paclitaxel [120]. In immunology, micelles have been studied as an adjuvant loading system or vaccine delivery system with promising results.

Micelles are much like dendrimers with only lipophilic substance encapsulation possible, low entity-loading capacity, and mean in vivo stability. Nonetheless, PMs can offer increased cargo solubility, improving its bioavailability. Furthermore, they can come with great control over drug release along with extended in vivo circulation time [187].

### 4.4. Inorganic Nanoparticles

Inorganic substances can be extremely toxic to our human body, although insignificant amounts can be tolerated. In fact, these elements are making their way up as promising career platforms for therapeutics as well as vaccines. Inorganic nanoparticles (INPs) come with rigorous control over structure and physicochemical properties for rigorous and precise drug delivery. INPs display different varieties of NPs. In the present review, we put together the most propitious in three categories.

#### 4.4.1. Gold NPs

Gold has been exploited for its putative medical properties throughout the history of civilisation. It was used in the early 20th century to help alleviate rheumatoid arthritis [191]. Recently, gold nanoparticles (GPN) have gained great interest as a transporter for pharmaceutical compounds or vaccines due to their plasmonic property, which could offer novel means of drug release. Surface plasmon resonance (SPR) is a resonance phenomenon caused by the interaction of metal NP conduction electrons with incident photons [190, 192].

**Figure 9:**
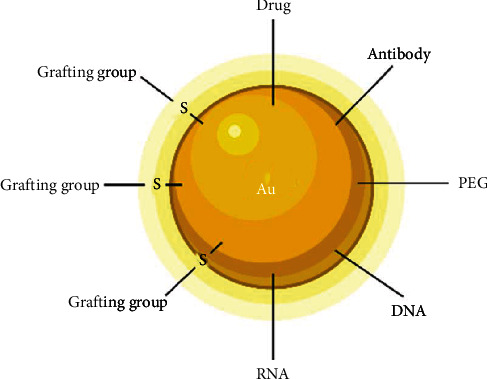
Schematic representation of a gold nanoparticle with different cargo possibilities.

Gold NPs are hybrids, displaying an inorganic core typically surrounded by an organic shell. The core governs the physical properties, while the chemical nature of the monolayer dictates the solubility and the reactivity of the particles [182, 183]. Subsequently, their size, pharmacokinetics, and physicochemical qualities can be meticulously tailored according to the need. AuNPs with a 10–150 nm size range are produced via reduction of gold salts with the presence of a stabilizing agent to prevent aggregation. More stable, monodisperse, and smaller NPs (6–8 nm) are achieved using recent advances and technology. Therefore, they are called gold nanoclusters [193]. Through interaction, gold may be directly coupled utilizing thiolated (-SH) molecules to produce stable monolayer-protected particles [194]. This thiol-mediated ligand-gold bond is highly stable in the extracellular environment. On the other hand, the monolayer is rapidly cleaved within the cell due to high glutathione levels providing a mechanism for internal release [194]. Targeted delivery is feasible with size control and surface functionalization using cell penetrating peptides and surface cell receptor ligands [194] as shown schematically in Figure 9. Understanding its pharmacokinetics is a fundamental step to develop a potent drug and vaccine delivery system. Au is masked by a hydrophobic monolayer immediately recognized by the RES and internalized by macrophages and other phagocytic cells, following administration. This could be very interesting in vaccine delivery, as the phagocytes navigate to lymph nodes and activate the immune system. Yet, this interaction displays a hard pitfall for drug delivery because of rapid elimination. In this manner, PEG functionalization is the key to skip this reticuloendothelial system uptake.

According to a study of size effect on GNP lymph node delivery and cytotoxic T-lymphocyte responses, the size threshold for the induction of potent cellular responses and T-cell polyfunctionality by GNPs lies between 10 nm and 22 nm [195]. AuNPs, conjugated with recombinant influenza haemagglutinin trimers and flagellin, showed enhanced and protective mucosal cellular immunity against the influenza virus [196]. Another intranasal formulation of AuNPs, conjugated with the M2e protein of influenza A, demonstrated protective immune response in mice against the H1N1, H3N2, and H5N1 influenza A viruses [167]. Unfortunately, no gold inorganic NP-based vaccines have been approved so far by the FDA. Nonetheless, AuNPs offer unprecedented characteristics as a delivery system, with high yield control of size, functionalization, drug release, and pharmacokinetics. One of AuNps' disadvantages is the tendency to accumulate in the liver and spleen, which may lead to toxicity. Coating with biocompatible materials can decrease their build-up and facilitate their elimination [133].

#### 4.4.2. Iron Oxide

Iron oxide (IO) is a chemical compound made up of iron and oxygen that is involved in a variety of geological and biological processes. Magnetite (Fe_3_O_4_), maghemite (g-Fe_2_O_3_), and hematite (-Fe_2_O_3_) are the three most prevalent forms of IO found in nature [197]. IO NPs have a similar structure design to AuNPs. In fact, their core, usually made of maghemite or magnetite, is layered by a biocompatible polymer, or less commonly, an inorganic coating [194, 198].

**Figure 10:**
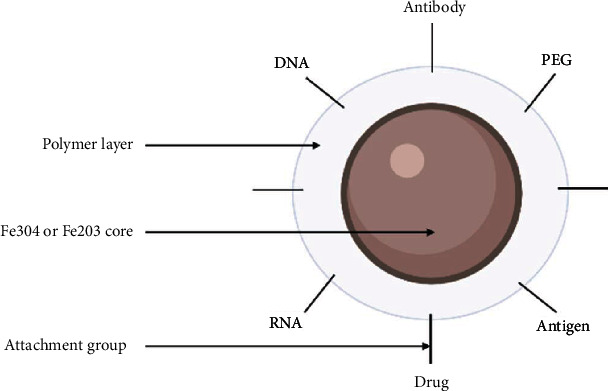
Schematic representation of an iron oxide.

The organic molecule surface modification serves many purposes: it stabilizes NPs in a biological solution, provides functional groups at the surface for subsequent derivatization, and prevents rapid absorption by the reticuloendothelial system (RES) [199, 200]. Formulated into nanostructures, they offer great biocompatibility, high surface volume ratio, low toxicity, and simple separation methodology. Furthermore, their superparamagnetic features display a simple targeted pharmaceutical delivery, via an external magnetic field applied near a specific organ or tissue. Therefore, they were intensely investigated as a drug delivery system and contrast agents for MRI [120]. SPIONs are created via a variety of physical, chemical, and biological methods. These techniques result in varied NP forms and, as a result, diverse characteristics [197]. Cargo can either be conjugated to surface moieties or encapsulated inside the particle outer layer or within coembedded mesoporous particles (Figure 10). For a synergistic combination with the iron oxide's hyperthermic characteristics, stimuli-responsive components for encapsulation can enable triggered cargo release [194]. In summary, IO NPs can be magnetically directed to a disease site, tracked via contrast imaging, heated at the affected sites, and provide triggered drug release [194]. With such rigorous control of drug delivery, IO NPs have been studied *in vitro* and *in vivo* for a wide range of applications in medicine, including as an MRI contrast agent and as a form of IV and intranasal [198] drug delivery, and for cancer treatment [201]. Within this framework, IO NPs' antiviral activity has been demonstrated *in vitro* [202], reporting their potential activity against influenza and SARS-CoV-2. A study by Abo-zeid et al. revealed that both Fe_2_O_3_ and Fe_3_O_4_ NPs interacted efficiently with the receptor-binding domain (RBD) of the SARS-CoV-2 S glycoprotein, leading to viral inactivation [203]. However, no SPION has been approved as a vaccine or antiviral drug delivery system; however, these nanostructures have been approved for intravenous iron replacement therapy in chronic kidney disease (Feraheme™/ferumoxytol; AMAG Pharmaceuticals) and as an MRI contrast agent (GastroMARK™; umirem®; AMAG Pharmaceuticals) [204]. This demonstrates the safety of IO, opening an avenue for another drug delivery system that needs to be well debriefed.

#### 4.4.3. Quantum Dots

Quantum dots (QDs) are tiny nanocrystals with a size range of 2–10 nm and are named for their shape. An inorganic core comprised of semiconducting elements like silicon, cadmium selenide, cadmium sulfide, or indium arsenide forms the architecture of these devices. This core has unique properties such as minimal tissue penetration, restricted light scattering, narrow emission bands, easy synthesis with a high surface-to-volume ratio, and low light scattering [205]. Consisting of hundreds to a few thousand atoms, the core of a QD is a layered structure with a metallic shell (i.e., ZnS) [206]. With their exceptional optical properties, QDs are able to reveal other NP delivery systems' biological reactions and pharmacokinetics. In fact, QDs can be loaded in other DDS with no effects on their characteristics or behaviours [206]. This system has been extensively explored as a theranostic for sensing, imaging, and therapy. As a drug carrier, they display enhanced bioavailability, controlled release, greater stability, and targeted delivery [118]. As an antiviral carrier, their antiviral activity was demonstrated against several viruses, such as SARS-CoV-2, pseudorabies virus, and respiratory syndrome virus [207, 208].

### 4.5. Self-Assembling Protein Nanoparticles and VLPs

#### 4.5.1. Self-Assembling Protein Nanoparticles (SAPNs)

Self-assembling protein nanoparticles (SAPNs) are structures with an expected size range from 20 to 100 nm. These proteins are constituted by coiled-coil domains composed of *α*-helical highly versatile amino acid sequences. The latter are characterized by seven-residue repeats, called heptad repeats (Hrs), in which polar residues and charged ones stabilize this unique formation with intramolecular interactions. Such interactions force the coiled-coil protein to self-assemble into nanoparticles [209]. Using a computational protein design, scientists were able to control the nanoparticle's size, shape, and characteristics and develop highly immunogenic epitopes that self-assemble into roughly spherical NPs [210]. As a consequence, we are able to deliver a cargo or display a repeated sequence of one or numerous antigens giving place to a strong immune response. Since 1981, a SAPN vaccine has been approved against the hepatitis B virus. The formulation was based on the surface antigen of the virus (HbsAg) that self-assembled into spherical particles with an average diameter of ~22 nm. Since then, numerous studies have been conducted to develop efficient SAPN-based vaccines against malaria, toxoplasmosis, HIV, SARS, and influenza [211]. The Malaria Protein 014 (FMP014) vaccine combined with a saponin molecule (ALF with QS-21) derived from the bark of the Quillaja species (ALFQ) reached phase 1 in clinical trials [212]. It was reported that immunization with a SAPN B-cell epitope of SARS-CoV-1 enhanced SARS neutralization *in vitro* and increased antibody titer production in mice [213]. Unfortunately, no further clinical trials have been conducted due to virus regression. Furthermore, a universal influenza vaccine was developed by Karch et al. with complete protection against lethal influenza A challenge in mice [214].

#### 4.5.2. Virus-Like Particles (VLPs)

**Figure 11:**
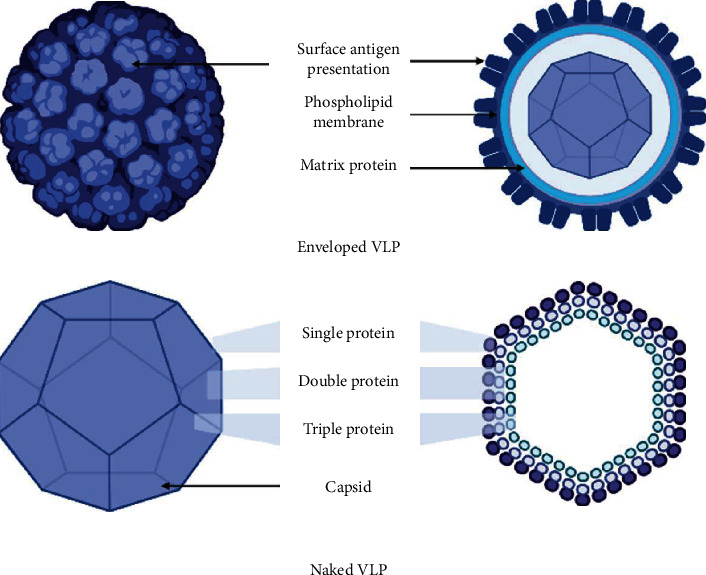
Schematic representation of virus-like particles.

Virus-like-particles (VLPs) are self-assembling polyprotein NPs, imitating the structure, organization, and the conformation of viruses with no genetic material as schematized in Figure 11. Therefore, these VLPs are noninfectious but efficiently internalized by antigen-presenting cells (APCs), and antigens can be presented with both major histocompatibility complex MHC classes 1 and 2, eliciting both humoral and cellular immune responses [215].

The structure can be made of a single or multiple capsid proteins covered by a cell membrane such as influenza VLPs or uncovered like papillomavirus VLPs. The production of such structures passes through injecting of recombinant plasmid with genes coding for the necessary structural protein in an expression system such as bacterial *E. coli*, yeast cell, baculovirus/insect cell, mammalian, and recently, plants [215].

VLPs range from 20 to 200 nm in size and are able to mimic the virus structure with the possibility of incorporating various types of antigens, giving broad spectrum coverage over highly mutational viruses, such as influenza. The possible addition of adjuvants confers an enhanced immune response. Vaccines against Human Papilloma Virus (HPV), such as Cervarix®, Gardasil®, and Gardasil9®, and vaccines against Hepatitis B Virus (HBV), such as the 3rd generation Sci-B-Vac™, are commercially available VLP-based vaccines [216]. A number of developed VLP vaccines are under clinical investigation (Table 1), and two of the most promising ones are Influenza and Norwalk. Chimeric VLPs [215] and Novavax's SARS-CoV-2 vaccine adjuvanted with their Matrix-M™ are currently in phase III clinical trials [73, 145, 170]. This proves the safety and efficiency of such nanovaccines for human use and gives researchers a promising alternative for vaccine formulation.

The VLP delivery system represents a cheaper and safer system for vaccination. A strong B-cell and T-cell response with no adjuvant is one of its assets in immunotherapy. As a result, VLPs trigger a protective response at low doses. However, their production can be tricky as separation is a complicated procedure. Another disadvantage of these DDS is the high cost of production and the sophisticated equipment required [217, 218].

## 5. Conclusions and Perspectives

Year after year, nanotechnology applied to drug/vaccine delivery has been proving itself. Nanoparticles are versatile systems, and researchers tend to carve their features depending on the unmet clinical need.

As a drug delivery system, nanoparticles were able to enhance cargo pharmacokinetics, safety, and efficacy. Pharmacokinetic-wise, the absorption of hydrophilic drugs such as many antiviral agents (efavirenz, aciclovir [219], and lopinavir) is greater when formulated in a nanostructure. For instance, oral administration of efavirenz loaded in PLGA nanoparticles had a 5.8-fold increase in the absorption than the marketed formulation [220]. As a matter of fact, nanoparticles are able to protect drugs from stomach acidity and they can facilitate uptake via transcytosis by M cells, epithelial cells, and Peyer's patches which results in higher bioavailability [221–223]. In regard to biodistribution, nanoparticles have achieved unprecedented control over substance release. Nanoformulations of acyclovir [219], zidovudine [224], efavirenz [225], and ribavirin [226] were associated with sustained release, while controlled and modulated release was attained with both nanoencapsulated ritonavir and lamivudine, under specific stimuli [227, 228]. Additionally, half-life is an important factor in drug efficacy and compliance. Usually, antiviral agents have a limited half-life which require daily multiple administrations. Nanoparticle-based drugs have shown greater and longer half-lives due to cargo protection against the chemical reactions of metabolism (ex. aebynin (coagulation factor IX), adynovate (antihemophilic factor), and arikayce (amikacin liposome inhalation suspension)) [229]. Safety is the first drug attribute to be evaluated in a phase I clinical trial. When side effects are frequent and significant, this turning point can limit the administration dose, and hence, treatment outcome. Safety is particularly improved via nanoparticle drug delivery systems. As an illustration, polymeric nanoparticle ribavirin has a lower possibility of accumulation in red blood cells compared to conventional ribavirin, and therefore, less haemolytic anaemia occurrence [230]. Use of nanoformulations was correlated with reduced toxicity and increased safety compared with conventional medicines. For instance, Curosurf (poractant alfa in a LNP), Doxil (doxorubicin HCl liposome), Abelcet, and AmBIsome (liposomal amphotericin B), DepoCyt (liposomal cytarabine), and Marqibo (liposomal vincristine) are all nanobased drugs that were granted approval by the FDA based on lower toxicity compared with conventional formulation counterparts [229]. All these examples show that nanoparticles can improve the benefit-to-risk ratio and could be useful for antiviral drug delivery in the context of RSVs.

Apart from small drug delivery, the interest of NPs has also been raised for the delivery of biotherapeutics. From the demonstration of antigen protection to vaccine application, there is only one step. Nanoparticles have therefore been proposed as vaccine drug delivery systems. The meticulous control over the structure, size, and chemical features of nanoparticles mediates their cell interactions and entrance. Uptake by dendritic cells might be facilitated with small NP size even though no difference related to the size was obtained regarding the immunological response in nonhuman primates [231]. In terms of composition, positively charged nanoparticles enhance the uptake of mRNA leading to a significant improvement of gene expression in mice [232]. The most recent example is the novel Pfizer/BioNTech and Moderna mRNA COVID-19 vaccines composed of lipid nanoparticles. These nanoobjects carry the mRNA, protect it from nuclease degradation, and allow its cell uptake. The role of the lipid carrier as adjuvant is another issue which should be carried out, as the charge alone might not be responsible for the adjuvanticity [233]. Nanoparticles can be functionalised with specific DC surface receptor (CD11c, CD40, and DEC205) ligands to improve their specific uptake. Moreover, their surface charge and composition can be tuned to increase their interactions and cell uptake, including mucosa/gut-associated lymphoid tissues. This is an interesting quality for mucosal vaccines (oral or nasal) against respiratory viruses. As a matter of fact, mucosal administration of vaccines comes with strong local immunity and production of specific IgA antibodies difficult to achieve with other vaccinal routes (IM and SC). Local IgA antibodies are able to opsonize the virus prior to cell entry; the first step of viral infection. Intranasal vaccination is an attractive route against respiratory viruses as it combines strong local immunity within the site of infection and systemic immunity. Table 1 contains multiple intranasal nanoparticle vaccines against RVs offering many key advantages.

Nanoparticles as vaccine delivery systems are promising innovations as they can simulate viral pathogens in structure and in a number of physical/chemical properties. All respiratory viruses have different architectures, antigens, and pathology pathways. Therefore, researchers are obligated to tailor nanoparticle features depending on cargo interactions and release needed.

Although NPs are of great interest, many challenges are confronted in their development. Characterization of these systems is a complicated process. Size, charge, architecture, and composition need to be well known and characterized. Multiple nanoplatforms with interindividual differences in formulations, in response to drug/vaccine delivery system requirements, make it hard to develop a standard nanoparticle. As a result, the safety and toxicity profiles of each novel nanoparticle developed need to be characterized to avoid unpredictable side effects. In addition, nanoparticle pharmacokinetics, structure-activity relation, and cargo loading and release are specific parameters for every DDS which needs to be well controlled and identified. The FDA through its Nanotechnology Characterization Lab (US-NCL) has provided protocols to standardize the characterization and evaluation methods to study nanoparticles dedicated to cancer [234].

The European counterpart, the EU-NCL, extended to health applications. These networks importantly contributed to the harmonization with issued guidance documents to translate more efficiently nanodrugs towards commercialisation. Related publications also showed the importance using several techniques to analyse more finally the nanoparticle shape and size distribution [235], highlighting also separation techniques which should be implemented prior to measuring the size distribution [236]. Industrial production of nanoparticles has also hampered their commercialisation. COVID-19 showed the difficulty of producing these vaccines at a large scale in a relatively short period of time. Of course, the management of this crisis is a strong future guidance for the next nanoparticles to be produced [231, 237] .

**Figure 12:**
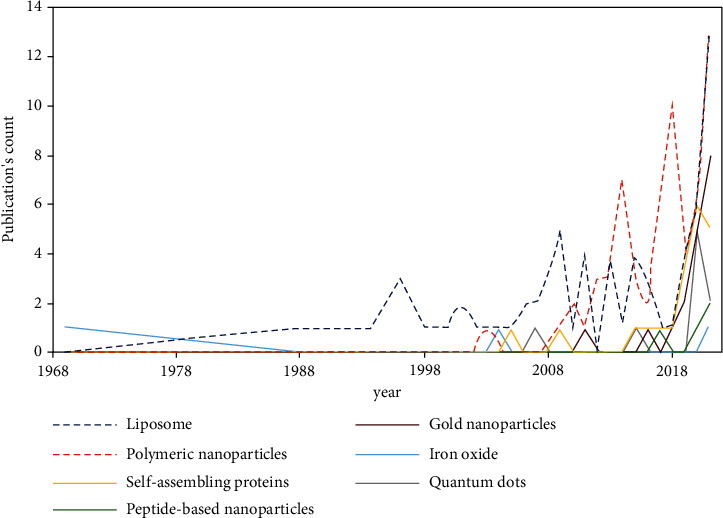
Number of publications related to RSVs according to the type of nanoparticles over a period of 50 years

From our survey and the reported data on Figure 12, we could see that liposomes were the earliest nanotechnologies reported in the literature followed by polymers; they are reported earlier than protein-based and inorganic-based nanoparticles and that all of them are increasing in the last few years. We can also comment on the fact that the amount of publications related to respiratory diseases still remains rather low as regard to the application of nanotechnologies for cancer. From the commercialization and production point of view, lipid-based nanoparticles are still ahead; however, as reported in Table 1 with polymers and the summarized expected results from phase 3 clinical trials with VLPs, we should also give worth consideration to these nanoparticles in the future. Therefore, the advances in both nanoparticle design, characterization, and scale-up, together with a better knowledge of the structure and mechanisms of virus entry, offer strong opportunities in the near future, to develop nanoparticles with additional features for RSVs. mRNA can easily be tuned to address various variants of the same virus. The design of lipids, polymers, and novel nanoparticulate structures will possibly allow tuning into the immune response level. As a prospect, the design of the next generation of nanoparticles in the field of vaccines and response to RSVs should be a combination of delivery and adjuvant properties. This is the current challenge of researchers in the field.

## Figures and Tables

**Table 1:** Nanoparticle-based vaccines against respiratory viruses.

Material	Size (nm)	Virus	Antigen/epitope	Adjuvant	Vaccine trial phase	Administration route	Benefits	References (year)
*Liposomes*								
DLPC liposomes	30–100	Influenza (H1N1)	M2, HA, and NP highly conserved peptides	MPL and trehalose 6,6′-dimycolate	In vivo Mice	Intranasal	(i) Protection against diverse influenza strains (ii) Highly specific T-cell responses that sharply limit viral replication following infection	[143] (2010)
Lipid nanoparticles (LPN)	43–54	Influenza (H1N1) (H3N2)	Hemagglutinin (split vaccine)	CpG-ODN (TLR 9 agonist)	In vivo Mice	Subcutaneous	(i) Improve adjuvant effects in vivo with greater production of cytokines and costimulatory molecules CD80 and CD86 on DCs (ii) Improved T-cell responses and protection over heterologous and heterosubtypic strain	[144] (2019)
	—	SARS-CoV-2	mRNA encoding full-length, prefusion stabilized spike protein	—	Licensed	Intramuscular (deltoid)	Safety, efficacy	[73, 145] (2021) Moderna
	—	SARS-CoV-2	Full spike mRNA	—	Licensed	Intramuscular (deltoid)	Safety, efficacy	[73, 145] (2021) BioNTech SE and Pfizer
	—	SARS-CoV-2	Spike mRNA	—	Phase 2b/3	Intramuscular	Safety, efficacy	[73, 145] (2021) CureVac
*Polymeric nanoparticles*								
PLGA	225.4	Bovine parainfluenza 3 virus (BPI3V)	BPI3V proteins	—	In vivo Calves	Intranasal	Greater mucosal IgA responses	[146] (2015)
	200–300	Swine influenza virus (H1N2)	Inactivated virus H1N2 antigen	—	In vivo Pigs	Intranasal	Reduce the clinical disease and induce cross-protective cell-mediated immune response in a pig model	[147] (2017)
*γ*-PGAa	100–200	Influenza (H1N1)	Hemagglutinin	—	In vivo Mice	Subcutaneous/intranasal	Sufficient cross-protective immune responses against influenza virus infection in mice Effective cross-protection with minimal side effects	[148] (2009)
PGA-PLL	150–600	RSV	G protein CX3C motif	—	In vivo Mice	Subcutaneous	Induce blocking antibodies that prevent the interaction of the RSV G protein with the fractalkine receptor (CX3CR1) and protect mice against RSV replication and disease pathogenesis	[149] (2015)
Chitosan	140	Influenza (H1N1)	H1N1 antigen	—	In vivo Mice	Intranasal	(i) Stimulate macrophages to produce IL-1*β* and IL-6 (ii) Stimulate spleen lymphocytes to produce IL-2 and IFN-*γ*	[150] (2015)
	220–500	All influenza viruses	HA2 and NP	Trimethyl chitosan TMC np	In vitro	—	(i) Systemic adaptive immunity (ii) Conserved proteins delivered in an adjuvanted nanoparticle system	[151] (2020)
	300–350	Influenza (H1N1)	HA-split	—	In vivo Mice	Intranasal	(i) Vaccine innocuousness (ii) Effective and safe delivery vehicle/adjuvant for the influenza vaccine	[152] (2014)
	571.7	Swine influenza virus (H1N2)	Killed swine influenza antigen	—	In vivo Pigs	Intranasal	(i) In CNP-KAg vaccinated pigs challenged with heterologous virus, reduced severity of macroscopic and microscopic influenza-associated pulmonary lesions were observed (ii) Chitosan SwIAV nanovaccine delivered by IN route elicited strong cross-reactive mucosal IgA and cellular immune responses in the respiratory tract that resulted in a reduced nasal viral shedding and lung virus titers in pigs	[153] (2018)
	200–250	Influenza (H1N1)	M2e	Heat shock protein 70c	In vivo Mice	Intranasal	(i) Induce a long lasting M2e-specific humoral and cellular immune responses (ii) Provide full protection against a 90% lethal dose (LD90) of the influenza virus A/PR/8/34 (H1N1).	[154] (2018)
HPMA/NIPAM	12–25	RSV	F protein	TLR-7/8 agonist	In vivo Mice	Subcutaneous	The improved pharmacokinetic profile by particulate polymer-TLR-7/8a was also associated with reduced morbidity and enhanced vaccine immunogenicity for inducing antibodies and T-cell immunity (i) Optimizing adjuvant design to elicit broad-based antibody and T-cell responses with protein antigens (ii) Protective immunity	[155, 156] (2018, 2015)
Polyanhydride	200–800	RSV	F and G glycoproteins	—	In vivo Calves	Intranasal	Enhanced interactions with antigen-presenting cells that are necessary in the initiation of efficacious immune responses BRSV-F/G nanovaccine is highly immunogenic, and with optimization, has the potential to significantly reduce the disease burden associated with RSV infection in both humans and animals	[157, 158]
*Self-assembling proteins and peptide-based nanoparticles*								
N nucleocapsid protein of RSV	15	RSV	RSV phosphoprotein	R192G detoxified *E. coli* enterotoxin LT	In vivo Mice	Intranasal	Efficient and safe intranasal vaccine against RSV	[159] (2008)
	15	RSV	FsII	Montanide™ Gel 01	In vivo Mice	Intranasal	N-specific cellular immunity and F-specific antibodies for protection	[160] (2016)
	15	Influenza (H1N1)	M2e	Montanide™ Gel 01	In vivo Mice	Intranasal	Nucleoprotein nanoring is a potent carrier for mucosal delivery of vaccine antigens	[161] (2013)
Ferritin	12.5	Influenza (H1N1)	M2e	—	In vivo Mice	Intranasal	High immunogenicity, cross-protection, and convenient administration, as well as being economical and suitable for large-scale production	[162] (2018)
Q11	—	Influenza (H1N1)	Acid polymerase	—	In vivo Mice	Intranasal	Immunogenic, noninflammatory, and promote more lung-resident memory CD8+ T cells compared to subcutaneous immunization	[163] (2018)
Self-assembling proteins	55	RSV	F protein (prefusion)	Squalene-oil-in-water	In vivo Mice	Subcutaneous	In mice and nonhuman primates, the full-valency nanoparticle immunogen displaying 20 DS-Cav1 trimers induced neutralizing antibody responses ∼10-fold higher than trimeric DS-Cav1	[164] (2019)
Protein recombinant nanoparticle ResVax	—	RSV	F protein	Aluminum phosphate	Phase III	Humans	First RSV vaccine to show Phase 3 efficacy Favorable safety and tolerability data	[165] (2020)- Failed to hit its primary endpoint
Self-assembling proteins		Influenza (H1N1, H3N2)	HA, M2e	—	In vivo Mice	Intramuscular	Controlled release Protective potency	[166] (2018)
*Inorganic nanoparticles*								
Gold	12	Influenza	M2e	CpG	In vivo Mice	Intranasal	Generate robust anti-M2e serum IgG antibodies in mice	[167] (2017)
Gold nanorods	21 × 57	RSV	F protein	—	In vitro	—	A potent method for immunizing against viruses such as RSV with surface glycoproteins that are targets for the human immune response	[168] (2013)-
Ferritin	—	RSV	F protein	AF03	In vivo Mice	Intramuscular	This pre-F vaccine increased the generation of NAbs targeting the desired pre-F conformation, an attribute that facilitates the development of an effective RSV vaccine	[169] (2020)-
*Others*								
VLP	—	SARS-CoV-2	Full-length, prefusion spike protein	Saponin-based Matrix-M™	Phase 3	Intramuscular	Safety	[73, 145, 170] (2021) Novavax
	80–120	Influenza (H1N1)	Hemagglutinin	—	In vivo Mice	Intranasal	Intranasal immunization with VLPs containing HA induced high serum and mucosal antibody titers and neutralizing activity against PR8 and A/WSN/33 (H1N1) viruses A promising strategy for the development of a safe and effective vaccine	[171] (2007)
	80–120	Influenza (H1N1, H3N2, and H5N1)	M2e	—	In vivo Mice	Intranasal	Cross-protection by inducing humoral and cellular immune responses	[172] (2018)
	—	Influenza (H3N2)	HA	Matrix-M1	Phase 1/2a	In vivo ferret	Broadly protective immunity and improved vaccine efficacy	[173] (2020)
	80–120	RSV	F protein and G glycoprotein of RSV and M1 protein of Influenza	—	In vivo Mice	Intranasal	Enhanced protection against live RSV challenges Significant decreases in lung viral replication and obvious attenuation of histopathological changes associated with viral infections	[174] (2017)
	—	RSV	RSV-F	Aluminum phosphate	Phase 3	Humans	(i) Tolerated without dose-related increases in adverse events (ii) A 7- to 19-fold increase in the anti-F IgG and a 7- to 24-fold increase in the antigenic site II binding and palivizumab competitive antibodies	[175] 2013 Not effective in a large phase 3 trial
	80–120	MERS-CoV	MERS spike protein (S)	Alum	In vivo Mice	Intramuscular	High-titer antibodies in mice	[176] (2014)
	80–120	MERS-CoV	Recombinant MERS-CoV S	Matrix-M1	In vivo Mice	Intramuscular	High-titer anti-S neutralizing antibody and protected mice from MERS-CoV infection in vivo	[177] (2017)
	80–120	MERS-CoV	MERS-CoV VLPs With protein S, E, and M	Alum	Rhesus macaques	Intramuscular	Excellent immunogenicity in rhesus macaques	[178] (2016)
ISCOMb	40	Influenza (H1N1)	Hemagglutinin	ISCOMATRIX	In vivo Sheep/mice	Intranasal	(i) Induce serum haemagglutination inhibition (HAI) titres in mice far superior to those obtained with nonadjuvanted vaccine delivered subcutaneously (ii) Induce mucosal IgA responses in the lung, nasal passages, and large intestine, together with high levels of serum IgA (iii) Improved protection	[179, 180] (2008, 2003)
Virosomes	150	Influenza A	Virus envelope proteins	Virosomes	Licensed	Intramuscular	Good immunogenicity in both healthy and immunocompromised elderly, adults, and children.	[181] (2009)

*Abbreviations*: DLPC—dilauroylphosphatidylcholine; MPL—monophosphoryl lipid A; CpG-ODN—unmethylated cytosine-guanine dinucleotide oligodeoxynucleotides; PGA—gamma-polyglutamic acid; PGA-PLL—poly(*γ*-glutamic acid)-poly(L-lysine); HA—hemagglutinin; NA—neuraminidase; IL—interleukin; HPMA—N-(2-hydroxypropyl)methacrylamide; NIPAM—poly(N-isopropylacrylamide); TLR—Toll-like receptor; ISCOM—immune stimulating complexes; SwIAV—swine influenza A virus; TMC np—trimethyl chitosan nanoparticle.

## Abbreviations

CDC: The Centers for Disease Control and Prevention
CoVs: Corona viruses
RVs: Respiratory viruses
RNA: Ribonucleic acid
mRNA: Messenger ribonucleic acid
DNA: Deoxyribonucleic acid
ssRNA: Single-stranded ribonucleic acid
ADV: Adenovirus
HAdV: Human adenovirus
HMPV: Human metapneumovirus
HboV: Human bocavirus
SARS: Severe acute respiratory syndrome
HIV: Human influenza virus
HPIV: Human parainfluenza virus
HRSV: Human respiratory syncytial virus
HRV: Human rhinovirus
ICTV: International Committee on Taxonomy of Viruses
LRTI: Lower respiratory tract infection
URTI: Upper respiratory tract infection
URT: Upper respiratory tract
IFN: Interferon
WHO: World Health Organization
IAV: Influenza A virus
NP: Nucleoprotein
MERS: Middle East respiratory syndrome
LNP: Lipid nanoparticle
RTI: Respiratory tract infections
NPC: Nuclear pore complex
ARTI: Acute respiratory tract infection
IDSA: Infectious Diseases Society of America
NAI: Neuraminidase inhibitor
RBV: Ribavirin
ICU: Intensive care unit
FDA: Food and Drug Administration
EMA: European Medicines Agency
NP: Nanoparticle
SUV: Small unilamellar vesicles
LUV: Large unilamellar vesicles
IND: Investigational new drug
LNP: Lipid nanoparticle
DC: Dendritic cell
PEG: Polyethylene glycol
siRNA: Small interfering RNA
PNP: Polymeric nanoparticle
PLGA: Poly(lactic-*co*-glycolic acid)
RES: Reticuloendothelial system
DDS: Drug delivery system
CS: Chitosan
TPP: Tripolyphosphate
PAMAM: Polyamidoamine
PPI: Polypropylene imine
VDS: Vaccine delivery system
CMT: Critical micelle temperature
CMC: Critical micelle concentration
INP: Inorganic nanoparticles
GNP: Gold nanoparticle
SPR: Surface plasmon resonance
AuNP: Gold nanoparticle
MRI: Magnetic resonance imaging
SPION: Superparamagnetic iron oxide nanoparticles
IV: Intravenous
IM: Intramuscular
SC: Subcutaneous
RBD: Receptor-binding domain
QDs: Quantum dots
VLP: Virus-like particle
SAPN: Self-assembling protein nanoparticle
MHC: Major histocompatibility complex
HPV: Human papillomavirus
HBV: Hepatitis B virus
Ig: Immunoglobulin
NCL: Nanotechnology Characterization Lab.
